# Eosinophilic meningitis epidemiological data from a national database in Thailand’s Department of Disease Control: a pragmatic, retrospective analytical study

**DOI:** 10.1186/s13023-022-02532-1

**Published:** 2022-10-27

**Authors:** Sittichai Khamsai, Verajit Chotmongkol, Somsak Tiamkao, Wanchai Maleewong, Panita Limpawattana, Watchara Boonsawat, Bundit Sawunyavisuth, Noppadol Aekphachaisawat, Kittisak Sawanyawisuth

**Affiliations:** 1 Department of Medicine, Faculty of Medicine, Khon Kaen University, Khon Kaen, Thailand; 2 Department of Medicine, Faculty of Medicine, Khon Kaen University, Khon Kaen, Thailand; 3 Department of Marketing, Faculty of Business Administration and Accountancy, Khon Kaen University, Khon Kaen, Thailand; 4 Central Library, Silpakorn University, Bangkok, Thailand

**Keywords:** *Angiostrongylus cantonensis*, Epidemiology, Thailand, Incidence, Mortality

## Abstract

**Background:**

Eosinophilic meningitis (EOM) is a rare neurological disease that can be misdiagnosed or underdiagnosed. Based on reported cases in the literature, there have been 2,827 cases worldwide since 1945. There are limited data on the prevalence and trends of EOM in a real-world setting, even in Thailand, the country with the highest prevalence of EOM. Therefore, this study aimed to evaluate the prevalence of EOM and EOM epidemiological data in a real-world setting.

**Methods:**

This was a pragmatic, retrospective analytical study using a national database. We retrieved EOM epidemiological data reported from government hospitals to Thailand’s Bureau of Epidemiology, within the Ministry of Public Health’s Department of Disease Control (DDC), between 2014 and 2019. The study was conducted by retrieving the data of all patients diagnosed with EOM and reported to the DDC. Diagnosis of EOM is made clinically by evidence of eosinophils of 10% or more of the total white blood cells in cerebrospinal fluid. Details of each patient were retrieved from the 506 Report Form, including age, month of reported case, zone of country, occupation, and mortality. Data regarding infection rate in each year and each zone were reported in rate/100,000 population, while data regarding age, month of reported case, and occupation were reported by year. Differences between means of age group, month of reported case, and occupation were tested by one-way analysis of variance (ANOVA). For those factors with significant differences among groups, Bonferroni method was used to compute pairwise differences.

**Results:**

There were 1,083 EOM cases reported in Thailand during the six-year study period. The average annual incidence of EOM was 180.5 cases, or 0.27 cases/100,000 population. The northeast zone had the highest rate, with 0.89/100,000 population. The common age groups were 25–54 years, with the highest rate among the 35–44 age group, with a mean of 38.3 persons/year. These age groups were significantly different from other age groups (F value 39.23; p < 0.001). A relatively high cumulative monthly incidence (> 100 cases) was seen in four months, including January (117 cases), September (103 cases), October (112 cases), and November (103 cases), though these rates were not significantly different from the other months’ rates. Regarding occupation, the top two occupations with EOM diagnoses were farmers and laborers, which were significantly different from other occupations (F value 99.95; p < 0.001). There was no reported case of death during the study period.

**Conclusion:**

EOM is common in Northeast Thailand among people of working age. The disease can be found throughout the year but is more common in the last quarter of the year. Farmers and laborers have the highest infection rate. To better understand the burden and outcomes of EOM, a national EOM reporting system with a better reporting form is required in endemic countries. Such a report form should include more details on risk exposure, symptoms, signs, treatment, and outcomes.

## Introduction

Eosinophilic meningitis (EOM) is a neglected and rare neurological disease found worldwide [1]. The most common cause of EOM is the parasitic infection *Angiostrongylus cantonensis* [2–4]. *A. cantonensis* is a roundworm neurotropic that was discovered by Chen in Guangzhou, China, in 1935 [5]. EOM in humans was first reported in 1945 and has been sporadically reported since then, with 2,827 reported cases in over 30 countries [1]. Among these countries, five had more than 100 reported cases in the literature, including Thailand, China, Tahiti, the USA, and Cuba.

Humans are infected with *A. cantonensis* via consumption of raw freshwater snails, shrimp, lizard, or contaminated water [3]. There are at least 51 molluscan families that serve as intermediate hosts for *A. cantonensis*, which is the main cause of EOM [6]. In Thailand and China, consumption of uncooked Pila snails is major transmission route for EOM, probably from regional eating habits [6]. The habit of eating raw snails, or “koi hoi,” has led to the highest numbers of reported EOM in Thailand [7].

Acute severe headache is a leading symptom of EOM [3, 4]. The mainstay treatment is corticosteroids, which aim to reduce headache [8]. In one study, the duration of headache was significantly reduced to five days with a 2-week course of corticosteroid, while the median duration of headache was 13 days and could last up to 56 days if left untreated [9]. Additionally, longer duration of headache may increase the risk of more severe encephalitis, leading to higher morbidity and mortality [10, 11].

Four previous reviews found that Thailand and China are the two main endemic countries, with several outbreaks each [1, 12–14]. One review found that Thailand had the highest number of reported cases, with 1,337 (47.33%), followed by China at 769 (27.22%) [1]. Countries in Asia Pacific also have reported cases in the literature, including the USA. Due to broad international travel, EOM cases are sporadically reported in Europe and Latin America [6]. There have been at least 77 reported cases in travelers and 22 reported cases in Europe [13, 14]. As review articles include only reported cases in literature [1, 12–14], there are limited data on EOM’s prevalence, trend, and epidemiological data in a real-world setting, even in Thailand, where EOM is most prevalent. Therefore, this study aimed to evaluate EOM’s prevalence and epidemiological data in a real-world setting, not only from the published data, to better understand the burden of disease to guide public health policy. Understanding the burden of disease could heighten the clinical index of suspicion in both endemic and non-endemic countries as EOM may be underdiagnosed or missed by physicians due to vague neurological signs, limited physician unawareness, or insufficient knowledge of this rare disease [14].

## Methods

This study was a pragmatic, retrospective analytical study using Thailand’s national database. We reviewed epidemiological data from the database of Thailand’s Bureau of Epidemiology, within the Ministry of Public Health’s Department of Disease Control (DDC) (http://doe.moph.go.th/surdata/disease.php?dcontent=old&ds=55). The study period was between 2014 and 2019.

The study was conducted by retrieving the data of all patients diagnosed with EOM and reported to the DDC. This report system is mandatory for all government hospitals and private hospitals, which are required to use the National Disease Surveillance form, also known as the 506 Report Form. Diagnosis of EOM is made clinically by evidence of eosinophils of 10% or more of the total white blood cells in cerebrospinal fluid. EOM is one of the diseases that is monitored at a national level, along with other communicable diseases such as anthrax, avian influenza, and leprosy. Details of each patient were retrieved from the 506 report form, including age, month of reported case, zone of country (north, northeast, central, or south), occupation, and mortality.

Descriptive statistics were used to show frequency and percentage. Average infection rate and mortality rate were reported. Infection rate data in each year and each zone were reported in rate/100,000 population, while data related to age, month of reported case, and occupation were reported by year. Differences between means of age group, month of reported case, and occupation were tested by one-way analysis of variance (ANOVA). For those factors with significant differences among groups, Bonferroni method was used to compute pairwise differences. Statistical analyses were executed by STATA software, version 10.1 (College Station, Texas, USA).

## Results

There were 1,083 EOM cases reported in Thailand during the six-year period. The average annual incidence of EOM was 180.5 cases, or 0.27 cases/100,000 population. The annual rate varied from 0.22 to 0.31/100,000 population (Table 1). The northeast zone had the highest rate, at 0.89/100,000 population.

**Table 1:** Showed infection rate of eosinophilic meningitis reported in Thailand between 2014 and 2019 (n = 1,083) by year and zone

Factors	Number (percentage)	Population	Rate/100,000 population
**Year**			
2014	204 (18.84)	64,955,313	0.31
2015	170 (15.70)	65,124,716	0.26
2016	175 (16.16)	65,426,907	0.27
2017	193 (17.82)	66,060,027	0.29
2018	197 (18.19)	66,301,242	0.30
2019	144 (13.30)	66,486,458	0.22
**Zone**			
North	85 (7.85)	61,494,004	0.14
Northeast	976 (90.12)	109,506,024	0.89
Central including Bangkok	10 (0.91)	110,442,845	0.01
South	12 (1.11)	46,425,332	0.03

The common age groups were 25–54 years (Table 2), with the highest rate among the 35–44 age group, with a mean of 38.3 persons/year. There were significant differences among age group by one-way ANOVA, with an F value of 39.23 (p < 0.001). The three age groups of 25–34, 35–44, and 45–55 years were significantly different from other age groups by Bonferroni method. However, these three age groups were not significantly different from one another (p > 0.05). The youngest age of EOM cases was < 1 year (3 cases). One case was reported from Chaiyaphum, Northeast Thailand, in 2016, and another was from Chiang Rai, North Thailand, in 2019.

**Table 2:** Showed numbers, mean (SD), and range of eosinophilic meningitis reported in Thailand between 2014 and 2019 (n = 1,083) by age groups

Age group/ year	2014	2015	2016	2017	2018	2019	Total	Mean, SD, range
**< 1**	0	0	1 (0.57)	0	0	1 (0.69)	**3 (0.28)**	**0.3, 0.5, 0–1**
**1–14**	12 (5.88)	11 (6.47)	8 (4.57)	6 (3.11)	9 (4.57)	8 (5.56)	**53 (4.89)**	**9.0, 2.2, 6–12**
**15–24**	26 (12.75)	27 (15.88)	25 (14.29)	30 (15.54)	29 (14.72)	18 (12.50)	**155 (14.31)**	**25.8, 4.3, 18–30**
**25–34**	31 (15.20)	24 (14.12)	41 (23.43)	38 (19.69)	42 (21.32)	23 (15.97)	**199 (18.37)**	33.2, 8.4, 23–42*
**35–44**	55 (26.96)	44 (25.88)	31 (17.71)	45 (23.32)	32 (16.24)	23 (15.97)	**230 (21.24)**	38.3, 11.7, 23–55*
**45–54**	46 (22.55)	32 (18.82)	33 (18.86)	47 (24.35)	39 (19.80)	32 (22.22)	**229 (21.14)**	38.2, 7.0, 32–47*
**55–64**	22 (10.78)	24 (14.12)	23 (13.14)	16 (8.29)	25 (12.69)	29 (20.14)	**139 (12.83)**	**23.2, 4.2, 16–29**
**65–74**	12 (5.88)	8 (4.71)	13 (7.43)	11 (5.70)	21 (10.66)	10 (6.94)	**75 (6.93)**	**12.5, 4.5, 8–21**
**> 75**	0	0	0	0	0	0	**0**	**0**
**Total**	**204 (100)**	**170 (100)**	**175 (100)**	**193 (100)**	**197 (100)**	**144 (100)**	**1083 (100)**	**180.5, 22.1, 144–204**

Note. *significant difference from other groups but not different among these three groups

A relatively high cumulative monthly incidence (> 100 cases) was seen in four months, including January (117 cases), September (103 cases), October (112 cases), and November (103 cases), as shown in Table 3. The average rates of monthly incidence of EOM in these four months were 19.5, 17.1, 18.6, and 17.1, respectively. There was no significant difference between months by one-way ANOVA (F value 0.165; p = 0.108).

**Table 3:** Showed numbers, mean (SD), and range of eosinophilic meningitis reported in Thailand between 2014 and 2019 (n = 1,083) by months

Months/year	2014	2015	2016	2017	2018	2019	Total	Mean, SD, range
**January**	22 (10.78)	21 (12.35)	12 (6.86)	16 (8.29)	11 (5.58)	35 (24.31 )	**117 (10.80)**	**19.5, 8.8, 11–35**
**February**	14 (6.86)	9 (5.29)	22 (12.57)	14 (7.25)	13 (6.60)	8 (5.56)	**80 (7.39)**	**13.3, 4.9, 8–22**
**March**	14 (6.86)	7 (4.12)	9 (5.14)	15 (7.77)	8 (4.06)	16 (11.11)	**69 (6.37)**	**11.5, 3.9, 7–16**
**April**	21 (10.29)	13 (7.65)	9 (5.14)	3 (1.55)	10 (5.08)	5 (3.47)	**61 (5.63)**	**10.1, 6.4, 3–21**
**May**	14 (6.86)	12 (7.06)	16 (9.14)	13 (6.74)	9 (4.57)	13 (9.03)	**77 (7.11)**	**12.8, 2.3, 9–16**
**June**	16 (7.84)	9 (5.29)	11 (6.29)	14 (7.25)	22 (11.17)	10 (6.94)	**82 (7.57)**	**13.6, 4.8, 9–22**
**July**	15 (7.35)	14 (8.24)	13 (7.43)	21 (10.88)	19 (9.64)	13 (9.03)	**95 (8.77)**	**15.8, 3.3, 13–21**
**August**	15 (7.35)	16 (9.41)	19 (10.86)	14 (7.25)	25 (12.69)	8 (5.56)	**97 (8.96)**	**16.1, 5.6, 8–25**
**September**	24 (11.76)	12 (7.06)	21 (12.00)	18 (9.33)	18 (9.14)	10 (6.94)	**103 (9.51)**	**17.1, 5.3, 10–24**
**October**	17 (8.33)	24 (14.12)	18 (10.29)	19 (9.84)	23 (11.68)	11 (7.64)	**112 (10.34)**	**18.6, 4.7, 11–24**
**November**	22 (10.78)	15 (8.82)	12 (6.86)	27 (13.99)	18 (9.14)	9 (6.25)	**103 (9.51)**	**17.1, 6.6, 9–27**
**December**	10 (4.90)	18 (10.59)	13 (7.43)	19 (9.84)	21 (10.66)	6 (4.17)	**87 (8.03)**	**14.5, 5.8, 6–21**
**Total**	**204 (100)**	**170 (100)**	**175 (100)**	**193 (100)**	**197 (100)**	**144 (100)**	**1083 (100)**	**180.5, 22.1, 144–204**

Regarding occupation, the top three occupations with EOM diagnoses were farmers, laborers, and students, with an average of 81.83, 49.67, and 24.50 cases/year, respectively (Table 4). There was significant difference among occupations by one-way ANOVA (F value 99.95; p < 0.001). Farmers and laborers were significantly different from other occupations (p < 0.001). Farmers were also significant differently from laborers (p < 0.001). There was no reported case of death during the study period.

**Table 4:** Showed numbers, mean (SD), and range of eosinophilic meningitis reported in Thailand between 2014 and 2019 (n = 1,083) by occupations

Occupation/year	2014	2015	2016	2017	2018	2019	Total	Mean, SD, range
**Farmers**	98 (48.04)	81 (47.65)	73 (41.95)	91 (47.15)	74 (37.56)	71 (49.31)	**488 (45.10)**	81.83, 10.46, 73–98*
**Laborers**	69 (33.82)	47 (27.65)	54 (31.03)	52 (26.94)	40 (20.30)	36 (25.00)	**298 (27.54)**	49.67, 11.71, 36–69*
**Students**	21 (10.29)	25 (14.71)	23 (13.22)	19 (9.84)	46 (23.35)	13 (9.03)	**147 (13.59)**	**24.50, 11.31, 13–46**
**Housemaids**	1 (0.49)	2 (1.18)	2 (1.15)	1 (0.52)	4 (2.03)	1 (0.69)	**11 (1.02)**	**1.83, 1.17, 1–4**
**Government officials**	1 (0.49)	0	0	1 (0.52)	3 (1.52)	1 (0.69)	**6 (0.55)**	**1.00, 1.10, 0–3**
**Merchants**	2 (0.98)	1 (0.59)	0	0	3 (1.52)	0	**6 (0.55)**	**1.00, 1.26, 0–3**
**Policemen/soldiers**	0	1 (0.59)	0	3 (1.55)	2 (1.02)	0	**6 (0.55)**	**1.00, 1.26, 0–3**
**Healthcare workers**	0	0	1 (0.57)	2 (1.04)	3 (1.52)	0	**6 (0.55)**	**1.00, 1.26, 0–3**
**Others**	9 (4.41)	5 (2.94)	3 (1.72)	1 (0.52)	2 (1.02)	4 (2.78)	**24 (2.22)**	**4.00, 2.83, 1–9**
**Unknown**	3 (1.47)	8 (4.71)	18 (10.34)	23 (11.92)	20 (10.15)	18 (12.50)	**90 (8.32)**	**15.00, 7.75, 3–23**
**Total**	**204 (100)**	**170 (100)**	**175 (100)**	**193 (100)**	**197 (100)**	**144 (100)**	**1083 (100)**	**180.5, 22.1, 144–204**

## Discussion

EOM had a steady prevalence over the six-year period, with the highest prevalence rate in Northeast Thailand. The common age groups were 25–54 years, and farmers and laborers were the two most common occupations. Note that the number of infected cases may be higher than reported in the literature (1,083 cases from 2014 to 2019 vs. 1,337 cases from 1945 to 2008) [1].

This study revealed that EOM is prevalent in Northeast Thailand, where consuming uncooked freshwater snails is a common eating habit. As previously reported, the most common age of EOM patients was in the third decade of life [4]. The youngest EOM patients were infants (Table 1). The transmission route for children may be from crawling or playing with contaminated snail or eating contaminated food or drink from parents’ hands [15–17]. As stated previously, people in Northeast Thailand commonly eat raw freshwater snails, particularly in the rice fields [7]. Thus, the highest incidence was found in farmers (45%), followed by laborers and students. The latter two occupations are believed to be infected from the same eating habit as farmers. Students in Northeast Thailand must work with their parents in the rice fields on weekends or semester breaks.

EOM in Thailand is reported throughout the year with a wax and wane pattern. The common months of EOM may be related, once again, to rice harvest season: September to January. As shown in Table 3, the numbers of EOM cases dropped in February to May, which is post-rice harvest. In Thailand, the rice harvest starts again in June and reaches the peak after September. An ecological study of EOM showed that the incidence of EOM cases was significantly related to wind speed negatively [18]. This rather peculiar phenomenon is explained as follows: strong wind reduces social and family sharing of food in the rice field. Additionally, it is a cultural habit of farmers to consume raw freshwater snails in the rice fields. Therefore, the prevalence of EOM may be highest in the rice harvest months [7]. Snails may reproduce more during the raining season as well.

During the study period, there was no case of death due to EOM. This finding may be explained by the definition of reported case. The 506 report form does not include the severe form of EOM, namely eosinophilic meningoencephalitis. This severe form has been found to have a high mortality rate: approximately 80% [10]. The conventional EOM reported in this study is less severe and may be self-limited, though it can take approximately two months to recover from severe headache. EOM patients may develop the severe form if they are left untreated or are misdiagnosed [11]. Ingestion of *A. cantonensis* larva by eating raw freshwater snails, shrimp, or contaminated salad may be possible transmission routes worldwide. To prevent this disease, providing education, washing produce, and refraining from consuming contaminated foods are crucial. In this report, sex data are not available on the website. From a previous report, cases among males are more prominent than among females, at a ratio of approximately 2–3:1, likely due to the higher frequency of males eating uncooked food [3, 8, 19].

Even though these data were collected only from Thailand, other countries with reported cases of EOM may replicate this study in their own setting to evaluate the epidemiological data. This study included data for six years. Data for the following six years will be reported, thus showing the trend of EOM.

There are some limitations in this study. First, the 506 report form does not include the severity of disease or the treatment provided. Second, patients with EOM who were misdiagnosis or who did not undergo a lumbar puncture may not be reported to the system. As mentioned earlier, EOM may be missed due to physicians’ lack of knowledge. Therefore, the prevalence indicated here may be under reported. Third, no causal relationship of personal factors or systematic review was studied [20–25]. Finally, there are no data regarding the total number of farmers in each zone or in Thailand overall.

## Conclusions

EOM is common in Northeast Thailand among people of working age. The disease can be found throughout the year but is more common in the last quarter of the year, and farmers and laborers have the highest infection rate. To better understand the burden and outcomes of EOM, a national EOM reporting system with a better reporting form is required in endemic countries. Such a report form should include more details on risk exposure, symptoms, signs, treatment, and outcomes.

## Data Availability

Data are available upon request to the corresponding author.
